# Isolated Congenital Facial Nerve Aplasia in a 13-Year-Old Child: Imaging Findings and Long-Term Functional Adaptation

**DOI:** 10.7759/cureus.105669

**Published:** 2026-03-22

**Authors:** Raj Barfa, Anita Mathew, Anushree R, Jitendra Sharma

**Affiliations:** 1 Radiodiagnosis, All India Institute of Medical Sciences, Bhopal, Bhopal, IND

**Keywords:** 3d ciss, congenital facial palsy, cranial nerve vii anomaly, facial nerve aplasia, internal auditory canal mri, neural plasticity

## Abstract

Facial nerve palsy at birth is an uncommon neurological condition that may arise from perinatal trauma or developmental abnormalities involving the facial nerve or its nucleus. While traumatic facial palsy is relatively common and often demonstrates spontaneous recovery, developmental causes such as facial nerve hypoplasia or aplasia are rare and usually associated with persistent deficits. In many cases, developmental facial palsy occurs as part of congenital syndromes involving multiple cranial nerves or systemic anomalies. Isolated absence of the facial nerve without associated syndromic features is extremely rare.

This case report presents the case of a 13-year-old child with isolated congenital left facial nerve palsy who presented with the inability to completely close the left eye and mild facial asymmetry since birth. High-resolution magnetic resonance imaging (MRI) demonstrated a complete absence of the facial nerve on the affected side. While most previously reported cases are described in infancy with limited evidence regarding long-term outcomes, this case demonstrates gradual functional adaptation despite congenital facial nerve absence.

## Introduction

Congenital facial paralysis is a rare disorder characterized by weakness or absence of facial muscle movement present at birth [1]. In most infants, the condition results from perinatal trauma, such as compression of the facial nerve during difficult or instrument-assisted delivery. It often presents as a transient neuropraxia that improves spontaneously over time. In contrast, developmental causes arise from abnormalities in the formation of the facial nerve or its brainstem nucleus during embryogenesis and typically lead to persistent facial weakness. These developmental abnormalities are frequently associated with syndromic conditions such as Moebius syndrome, Poland syndrome, and Goldenhar syndrome, where facial nerve dysfunction occurs alongside other craniofacial or systemic anomalies [2]. However, isolated congenital absence (aplasia) of the facial nerve without accompanying syndromic features is extremely rare [3].

We report a case of isolated congenital left facial nerve aplasia in a 13-year-old child, emphasizing the diagnostic role of magnetic resonance imaging (MRI) and the long-term functional adaptation observed in longstanding facial nerve deficiency.

## Case presentation

A 13-year-old male child presented with a history of inability to close the left eye and facial asymmetry that had been present since birth. According to parental history, weakness of the left side of the face in the form of inability to close the left eye and deviation of the mouth toward the right, particularly evident during crying, was more severe in infancy.

The antenatal period was uneventful, and there was no history of maternal illness, drug exposure, or complications during pregnancy. Delivery was performed via cesarean section for cephalopelvic disproportion. The newborn had normal Apgar scores and an appropriate birth weight. There was no history of birth trauma, forceps-assisted delivery, or perinatal asphyxia. Hearing screening performed during infancy was normal.

**Figure 1:**
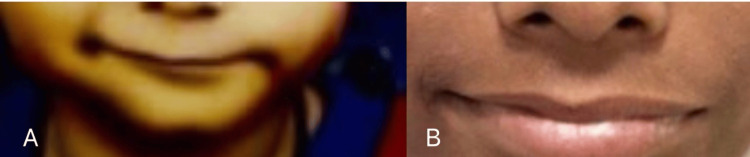
Clinical images demonstrating improvement in facial asymmetry over time. (A) Early childhood image showing rightward deviation of the mouth with absence of the left nasolabial fold. (B) Follow-up image at present showing significant improvement with subtle facial asymmetry.

**Figure 2:**
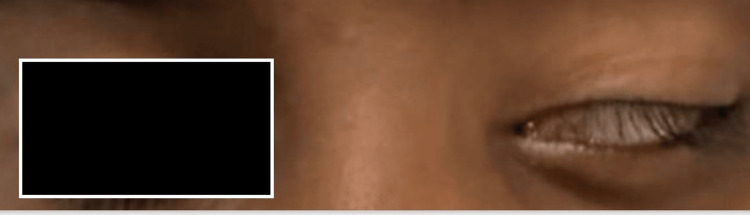
Present clinical image showing incomplete closure of the left eye

During early childhood, the facial asymmetry showed gradual improvement with subtle asymmetry at present (Figure 1). There is mild, persistent, incomplete eyelid closure on the affected side; however, it has also decreased in severity (Figure 2). According to the House-Brackmann grading, facial palsy was grade V at birth and grade IV at present. The child showed normal growth and age-appropriate motor, cognitive, and social development. There was no history of recurrent ear infections, hearing impairment, or speech delay. Family history was unremarkable for congenital anomalies or neurological disorders.

Examination of the external ears, auditory canals, and tympanic membranes was normal. No abnormalities of the pinna or external auditory canal were identified. Detailed neurological examination revealed no involvement of other cranial nerves. There were no dysmorphic facial features, skeletal anomalies, or neurocutaneous markers suggestive of a syndromic disorder.

Imaging findings

**Figure 3:**
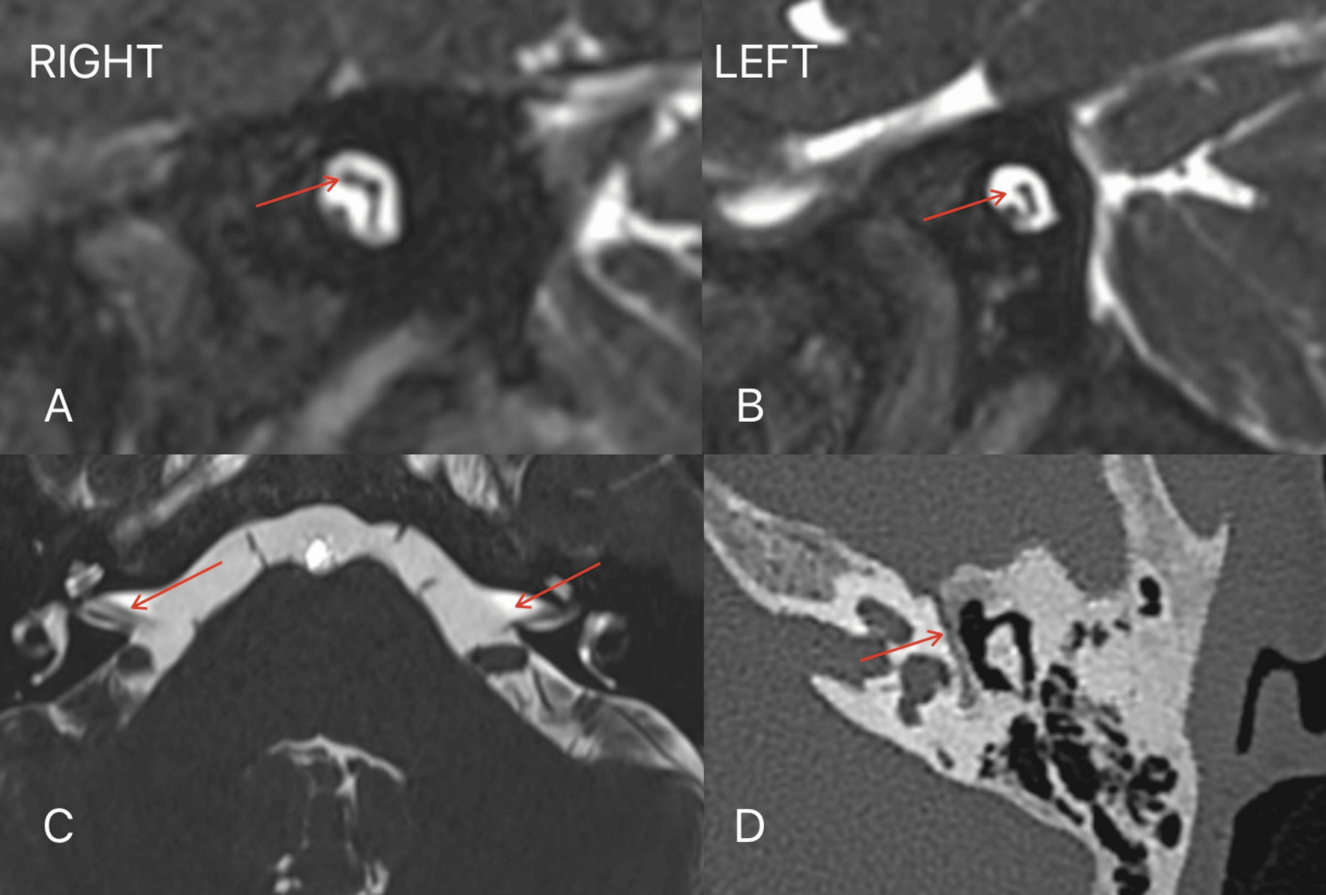
(A) Oblique sagittal 3D CISS MRI sequence along the right internal auditory canal (IAC) - normal right facial nerve in the anterosuperior quadrant of the right IAC (arrow). (B) Oblique sagittal 3D CISS MRI sequence along the left IAC showing an empty anterosuperior quadrant of the left IAC (arrow) with a comma-shaped vestibular nerve complex. (C) Posterior fossa axial 3D CISS MRI sequence showing normal cisternal segment of the right facial nerve (arrow) with absence on the left side. (D) High-resolution CT showing a normally developed bony left facial canal (arrow). CISS: Constructive interference in steady state.

High-resolution computed tomography (CT) (Figure 3, Panel D) of the temporal bones was performed to evaluate the osseous structures of the ear and facial nerve canal. CT demonstrated normal morphology of the external, middle, and inner ear structures. The internal auditory canals were symmetrical and of normal caliber, and no bony abnormalities were identified.

Subsequently, MRI of the brain and internal auditory canals was performed using high-resolution heavily T2-weighted three-dimensional (3D) constructive interference in steady state (CISS) sequences in axial and oblique sagittal planes perpendicular to the seventh and eighth nerves. In the axial plane (Figure 3, Panel C), along the posterior fossa, a complete absence of the cisternal segment of the left facial nerve was revealed. At the same time, it was normally seen on the right side. The oblique sagittal plane (Figure 3, Panel B) showed the absence of the facial nerve in the anterosuperior quadrant of the left internal auditory canal (IAC). The cochlear, superior, and inferior vestibular nerves were clearly visualized and appeared normal in size and morphology.

The brainstem, cerebellopontine angles, and intracranial structures were otherwise normal. Bilateral inner ear structures, including the cochlea, vestibule, and semicircular canals, showed normal anatomy. These imaging findings were consistent with isolated left facial nerve agenesis.

## Discussion

Congenital facial nerve aplasia is an exceptionally rare developmental anomaly marked by the complete absence of the facial nerve, which manifests as facial muscle weakness typically observed at birth or shortly thereafter [4]. While this represents a structural deficit, it must be distinguished from the broader clinical presentation of congenital facial palsy (CFP). Structural abnormalities like aplasia do not cause most cases of CFP but are instead attributed to perinatal trauma, such as mechanical compression or difficult labor, rather than developmental agenesis [2,3].

Congenital facial paralysis represents approximately 8%-14% of pediatric facial paralysis cases, with an overall incidence of 0.8-2.1 per 1000 live births, most commonly related to birth trauma during difficult labor (88%), particularly forceps-assisted delivery. Less commonly, developmental etiologies such as Moebius syndrome, congenital unilateral lower lip palsy, and inherited myopathies like facioscapulohumeral muscular dystrophy may be implicated [1].

Perinatal or traumatic facial palsy most frequently occurs in the setting of complicated labor, intrauterine malposition, or external compression during delivery [2]. Mechanical factors such as the use of obstetric instruments, prolonged labor, or pressure against the maternal pelvic structures can temporarily affect the facial nerve [5]. In most instances, this type of palsy reflects transient neuropraxia, and affected infants demonstrate spontaneous recovery within several weeks to months as nerve function gradually returns [2,3].

In contrast, developmental causes are related to abnormalities in the formation of the facial nerve, its brainstem nucleus, or disruptions during embryological development of the cranial nerve pathways [6]. These abnormalities often result in persistent facial weakness that does not resolve spontaneously. Developmental facial palsy may occur in isolation or may be associated with other structural or syndromic anomalies [4,7].

Several congenital syndromes have been reported in association with developmental facial nerve abnormalities. Among the most recognized is Moebius syndrome, which involves congenital paralysis of both the sixth and seventh cranial nerves, leading to impaired facial expression and restricted ocular movements. Another condition is Poland syndrome, in which there is unilateral absence or hypoplasia of the pectoralis major muscle accompanied by ipsilateral upper limb anomalies. Additionally, Goldenhar syndrome, also known as the oculo-auriculo-vertebral spectrum, may present with craniofacial asymmetry, ear anomalies, vertebral defects, and facial nerve dysfunction [2,7,8].

In rare instances, such as in the present case, developmental facial palsy may occur without any associated syndromic features, resulting from isolated structural absence or severe hypoplasia of the facial nerve itself [3,9]. Only a few cases have been reported in the literature since the first description by Jervis and Bull [9]. Most published reports are isolated case reports, with the largest small series described by Decraene et al. [10]. Long-term clinical follow-up is rarely documented, making cases with extended follow-up particularly uncommon and valuable for understanding the natural history and functional adaptation over time.

MRI is the imaging modality of choice for evaluating the cisternal and intracanalicular segments of the facial nerve. With advances in high-resolution MRI, particularly heavily T2-weighted three-dimensional sequences such as 3D-CISS, direct visualization of the cranial nerves within the cerebellopontine angle and IAC has become possible. These imaging techniques play a crucial role in confirming the diagnosis by demonstrating the absence of the facial nerve and excluding other structural abnormalities. Recognition of this entity is important for differentiating developmental facial palsy from traumatic causes, guiding prognosis, and assisting in appropriate clinical counseling and management [4,11].

CT of the temporal bone primarily evaluates osseous anatomy and may appear normal even in the presence of facial nerve agenesis. Therefore, a normal CT scan does not exclude developmental abnormalities of the facial nerve. MRI is essential for definitive diagnosis [12].

Non-traumatic congenital facial nerve palsies generally have a poor functional prognosis [13]. An interesting feature of the present case is the improvement in facial movements observed over time despite the absence of the facial nerve. This phenomenon may be explained by neural plasticity and compensatory reinnervation from adjacent cranial nerves. Aberrant innervation from the trigeminal or hypoglossal nerves has been proposed as a possible mechanism contributing to partial functional recovery [4].

## Conclusions

Isolated facial nerve agenesis is an exceptionally rare cause of facial palsy present since birth. High-resolution MRI plays a crucial role in demonstrating the absence of the facial nerve and differentiating developmental anomalies from traumatic causes of facial palsy. Recognition of this condition is essential for accurate diagnosis, appropriate counseling, and long-term management planning. This case also highlights the potential for partial functional adaptation over time despite congenital absence of the facial nerve.
